# *In Vitro* retinal ganglion cell differentiation and enrichment under the scope: do subtypes matter?

**DOI:** 10.3389/fcell.2025.1750142

**Published:** 2026-02-16

**Authors:** Tahani W. Baakdhah, Jeremy M. Sivak

**Affiliations:** 1 Donald K Johnson Eye Institute, Krembil Research Institute, University Health Network, Toronto, ON, Canada; 2 Department of Ophthalmology and Vision Science, University of Toronto School of Medicine, Toronto, ON, Canada; 3 Department of Laboratory Medicine and Pathobiology, University of Toronto School of Medicine, Toronto, ON, Canada

**Keywords:** glaucoma, pluripotent stem cells, retinal ganglion cells, retinal injury, retinal organoids, retinal regeneration

## Abstract

Retinal ganglion cells (RGCs) play a pivotal part transmitting visual data to the brain. Yet, damaged RGCs are unable to maintain and regrow axons and connectivity, as in the common blinding disease glaucoma. Thus, the idea of rescuing and replacing damaged RGCs holds immense therapeutic potential. In recent years pluripotent stem cells cultured in both 2D and 3D (retinal organoid) environments have generated RGCs from healthy- and patient-derived cells. These models can be used to study normal retinal physiology and compare it to the diseased retina. Although the effects of glaucomatous injuries on RGCs have been well-studied in animal models, much less is known about similar mechanisms in the human retina. Further, using *in vitro*-derived RGCs as a tool for cell characterization and replacement is still in its infancy. In particular, many distinct RGC subtypes have been described, and it remains unclear how well this diversity is reflected in the various differentiation protocols, or their functional roles in human health and disease. In this review we summarize the currently described subtypes of human RGCs and their markers and discuss recent evidence for subtype-specific vulnerabilities to injury and disease. Finally, we synthesize the limited evidence for subtype differentiation in human stem cell culture approaches. Increased understanding of this human RGC diversity will provide new tools to enrich for selective subtypes and ultimately fill key translational gaps in human glaucoma research.

## 1 Introduction

**FIGURE 1:**
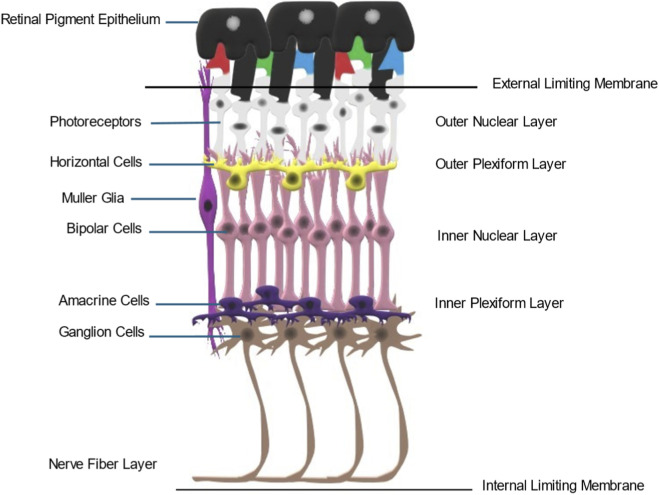
Structural organization of the mammalian retina. A cartoon depicting major retinal cell types and their architectural organization. Retinal ganglion cells are located within the deepest retinal cell layer. They receive inputs from amacrine and bipolar cells, which gather visual information from the rod and cone photoreceptors. The ganglion cell axons form the nerve fiber layer and exit the retina at the optic nerve head to form the optic nerve, which projects to several targets in the midbrain.

RGCs are a heterogeneous population of neurons that play a crucial role by transmitting visual information from early processing in the retina to the brain (Levin, 2005; Kerschensteiner and Feller, 2024) (Figure 1). They are responsible for encoding various aspects of visual stimuli, such as color, shape, and motion (Yan et al., 2020). Certain subtypes of RGCs also have indirect visual functions, such as regulating the size of the pupil in response to changes in light levels. Unlike photoreceptors, which are comprised of only four major human subtypes, with often only a single synaptic connection, RGC diversity is much more complex.

**FIGURE 2:**
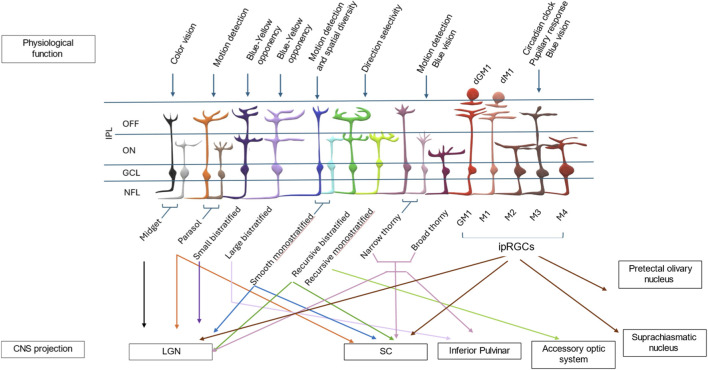
Major RGC subtypes identified in the human and non-human primate retina. A cartoon depicting the most studied human and non-human primate RGC subtypes, their dendritic stratifications into the IPL, associated physiological functions (labeled above each subtype), and projection pathways to CNS targets (indicated by arrows below). (INL, inner nuclear layer; IPL, inner plexiform layer showing ON and OFF sublaminae; GCL, ganglion cell layer; NFL, nerve fiber layer; GM1, gigantic M1 ipRGC; dGM1, displaced gigantic M1; dM1, displaced M1; LGN, lateral geniculate nucleus; SC, superior colliculus.)

Primates and non-primates alike possess many RGC subtypes that differ from each other in multiple ways, including: molecularly and biochemically (e.g., having a diverse gene expression profile) (Kang et al., 2017; Mead et al., 2014; Polyak and Willmer, 1949; Yan et al., 2020), morphologically (i.e., cell soma size and/or stratification) (Dacey, 2004; Poljak, 1935), and physiologically (e.g., some respond to movement while others respond to light, or to regulate circadian rhythms) (Gouras, 1968; Graham et al., 1978; Johnson and Samuels, 1997; Kuffler, 1953; Pokorny, 2011; Stiles, 1959). These functionally and topographically unique subtypes of RGCs receive afferent input from complex inner retinal circuits that can include dozens of presynaptic bipolar and amacrine cells. All RGCs then extend lengthy axons through the optic nerve and into one of several visual centres in the brain. By combining these features, RGCs have been classified into multiple distinct subtypes that can respond differently to injury or disease (Figure 2). Recent single-cell RNA sequencing (scRNA-seq) breakthroughs have revolutionized subtype mapping, revealing gene expression profiles that delineate functional classes. For instance, in mice, comprehensive scRNA-seq atlases have identified over 40 subtypes, characterized by markers like *SPP1*/*KCNG4* for alpha RGCs (motion-sensitive) and *OPN4* for ipRGCs (regulating circadian rhythms), enabling spatial and developmental insights through integration with spatial transcriptomics (Budoff and Poleg-Polsky, 2025; Li et al., 2024). In primates (including humans), scRNA-seq has uncovered 18–25 subtypes. Cross-species comparisons have highlighted evolutionary divergences, such as enriched midget RGCs for foveal high-acuity color vision in primates versus broader motion-detection subtypes in nocturnal mice (Yi et al., 2021; Lu et al., 2020). These transcriptomic atlases, including multi-species datasets and aging-focused profiles, underscore species-specific adaptations. Mouse studies leverage advanced genetic tools for finer classification, while human/primate studies face tissue access limitations but benefit from emerging databases like scRetinaDB, aggregating over 2.79 million cells across species for subtype vulnerability analysis in diseases (Tang et al., 2025).

Adding additional complexity, this diversity of subtypes is species-specific. Distinct RGC subtypes have been identified in humans in comparison to mice. Curiously, the mouse has many more identified subtypes when compared to humans (more than 40 subtypes in the mouse eye, compared to about 18 in the human eye) (Corral-Domenge et al., 2022; Rheaume et al., 2018; Sanes and Masland, 2015; Tran et al., 2019; Yamada et al., 2005). The higher number of RGC subtypes in mice compared to primates likely stems from evolutionary adaptations and differences in research methodologies. From an evolutionary perspective, mice are nocturnal animals that lack a specialized fovea. They may require a broader array of specialized cell subtypes to perform complex computational visual processing, such as motion and looming detection, across a uniform retina. These adaptations facilitate rapid, innate survival behaviors. In contrast, primates prioritize fewer specialized types for high-acuity trichromatic vision, with a retina dominated by midget cells that act as “pixel encoders.” These cells transmit high-resolution, relatively raw data to the brain, shifting the burden of complex visual processing from the retina to the visual cortex. Furthermore, the current disparity between species is reinforced by methodological asymmetries. The availability of sophisticated transgenic mouse lines and high-throughput transcriptomic tools allows for the identification of extremely rare murine RGC subtypes that comprise less than 1% of the population. In the primate retinas, the overwhelming numerical dominance of midget and parasol cells, combined with more limited access to similar genetic tools, makes the characterization of rare subtypes significantly more challenging, potentially undercounting the true diversity (Kim et al., 2021; Hahn et al., 2023; Huang et al., 2024).

Considering this cellular and morphological complexity, the preservation or replacement of damaged RGCs due to injury or degenerative disease remains a major challenge. Yet, irreversible RGC degeneration drives vision loss in glaucoma; a leading cause of blindness worldwide (Alqawlaq et al., 2019; Weinreb et al., 2016; Almasieh et al., 2012). Addressing this important issue requires understanding how specific subtypes function and respond to stress and injury, followed by methods for the therapeutic manipulation of cellular pathways involving neuronal survival, migration, dendritogenesis and axogenesis, pathfinding, synaptogenesis, and myelination. These data will also facilitate the development of protocols to enrich and/or differentiate for specific subtypes, particularly those of the human retina. Recent research has been pioneering use of *in vitro* models to grow human retinal neurons, including RGCs, from human embryonic stem cells (ESCs) and induced pluripotent stem cells (iPSCs). However, the isolation of and study of RGC subtypes from these models is still unclear. To date, labs have tended to use either pan-RGC markers (expressed by all subtypes) or mouse-specific markers that may not have the same specificity in human cells. Yet, there is increasing published information concerning human RGC subtype identification and roles, and their responses to injury and disease. To comprehensively address this point, this review will: 1) explore the different subtypes of human RGCs, 2) profile relevant human molecular markers available for identifying and isolating each subtype, 3) evaluate evidence for subtype vulnerability in injury and disease, and 4) assess current strategies for subtype differentiation in human PSC (hPSC) cultures, leveraging transcriptomic insights for therapeutic translation.

## 2 Retinal ganglion cell subtypes in humans

Unlike in other species, the identification of RGC subtypes in humans is faced with many hurdles. Acquiring eyes donated from different age groups and healthy or sick individuals is challenging, as most samples are collected *postmortem*. Using these tissues, researchers have tried to understand the development of human RGCs and their specification in the retina as well as their connections to the brain’s visual cortex. Sectioning and staining these human samples to identify subtype-specific molecular markers is still a technical challenge. In addition, testing the physiological response in living or *postmortem* eyes is extremely difficult and often unreliable. One alternative approach has been to use non-human primates as an RGC model to study physiological responses of different subtypes. Clinically, optical coherence tomography (OCT) imaging has provided additional tools to define changes in RGC and nerve fiber architecture, and the pathological progression of inherited and acquired optic neuropathies (Kim et al., 2021).

There are about 18 peripheral and 16 central RGC subtypes in primates (Dacey, 2004; Masri et al., 2019; Peng et al., 2019; Rheaume et al., 2018; Yan et al., 2020). Of these, the three major RGC subtypes are: ON and OFF Midget RGCs, ON and OFF parasol RGCs and small bistratified RGCs (Grünert et al., 2021; Lee et al., 2010; Merigan and Maunsell, 1993). The remaining subtypes include recursive bistratified, recursive monostratified, broad, and narrow thorny, smooth monostratified, large sparse, and giant sparse melanopsin ganglion cells (Ghosh et al., 1996; Grünert et al., 2021; Hughes et al., 2016; Kim et al., 2021; Mao et al., 2014). Interestingly, in primates the distribution of RGC subtypes appears to be spatially organized. For example, of the total RGCs in the human fovea, midget cells constitute about 90%, parasol cells about 5%, and small bistratified cells about 1%. In the periphery, midget cells make up about 40%–45%, parasol cells about 15% and small bistratified cells about 10% of the total. (Dacey, 1994; Field et al., 2007; Masri et al., 2019; Peng et al., 2019; Vogelsang et al., 2025; Zhang et al., 2024). Each of these subtypes has received varying degrees of research and so range from well-studied to virtually unknown. Known classification and roles for various primate RGC subtypes are described in Table 1 and in the section below.

**TABLE 1:** Distribution and proportion of identified RGC subtypes in the primate retina. The estimated relative abundance of major and rare RGC subtypes identified in the primate retina. Proportions are expressed as a percentage of the total local RGC population.

RGC subtype	% Total retina	% In macular (central/Foveal)	% In peripheral	References
Midget	70%	90%–95%	40%–45%	Kim et al. (2021), Hibble et al. (2025), Dacey (1993); Yamada et al. (1996)
Parasol	10%	5%	15%	Crook et al. (2014), Jacoby (1998), Ma et al. (2023)
Small bistratified	5%–8%	1%	10%	Masri et al. (2019), Dacey (1994)
Large bistratified	2%–3%	<1%	2.5%–4%	Zheng et al. (2024), Peng et al. (2019)
Smooth monostratified	1.5%	<0.1%	1%–2%	Petrusca et al. (2007), Peng et al. (2019)
Recursive RGCs Monostratified	2%–3%	<1%	2.5%	Patterson et al. (2022), Hahn et al. (2023)
Bistratified	1.5%	<0.5%	1.5%	Detwiler et al. (2019), Kim et al. (2022)
Thorny RGCs	1%	0.1%	1%–2.5%	Grünert and Paul (2020), Kim et al. (2022)
ipRGCs	0.2%–1.5%	<0.01%	1%–1.5%	Mure (2021), Liao et al. (2016), Hannibal et al. (2017)

### 2.1 Human RGC subtypes

#### 2.1.1 Midget RGCs

This subtype is also known as parvocellular RGCs (P cells) and account for 70% of primate RGCs. These cells are concentrated in the central retina and project to the parvocellular layer of the lateral geniculate nucleus (LGN) (Dacey, 1993; Yamada et al., 1996). Midget RGCs has small cell bodies with small dendritic fields (5–10 μm in diameter in the central retina and up to 225 μm in the periphery), which correspond to smaller receptive fields than those of other RGCs. In the central retina, midget RGCs have a one-to-one connectivity with midget bipolar cells that draw their input from a single cone (Kling et al., 2019). However, at the peripheral retina, midget RGCs have larger dendritic fields and more than one midget bipolar axon synapses with the dendritic tree of the receiving peripheral midget ganglion cell. There are two types of midget RGCs: the outer stratified OFF-midget cells show smaller dendritic fields and higher cell densities than the inner ON-midget cells (Dacey, 2000). Cells branching in sublamina **a** will be OFF centre and those branching in sublamina **b** will be ON centre. Thus, the midget ganglion cells branching close to the amacrine cell layer will be OFF centre and those branching close to the ganglion cell layer will be ON centre (Nelson et al., 1978). They are involved in red and green vision through connecting to M and L bipolar cells and M and L cones. ON-midget cells will connect to either red ON/green OFF or to green ON/red OFF cones. OFF-midget cells will connect to red OFF/green ON or to green OFF/red ON cones. This conclusion is derived from both physiological evidence (Martin et al., 2001; Reid and Shapley, 1992) and reported functional connectivity (Field et al., 2010). However, recent studies suggest that some OFF-midget cells receive signals from short wavelength (blue) sensitive cones (Tsukamoto and Omi, 2015; Wool et al., 2019). In addition to color discrimination, midget RGCs also transmit pattern, texture and stereoscopic depth perception information (Schiller, 2010). To summarize, two midget RGC types identified to date, OFF-midget and ON-midget, aid in color vision, pattern recognition and depth perception through organized connectivity.

#### 2.1.2 Parasol RGCs

Also known as the magnocellular RGCs or M-cells (equivalent to alpha cells in mice and Y cells in cats), they account for approximately 5% of all ganglion cells in the central retina and 15% in the peripheral retina (Crook et al., 2014; Jacoby, 1998; Ma et al., 2023). They project to the magnocellular layer 1 and 2 of the LGN and the superior colliculus (SC) (Ma et al., 2023). In comparison to midget cells, parasol RGCs have larger receptive fields and cell bodies. They are more sensitive to luminance contrast than midget cells and respond more transiently to light stimuli. They have a greater absolute sensitivity to light than midget cells because they receive signals from a larger area of the retina and more input from the rod pathway (Kaplan and Shapley, 1986). As with midget cells, there are two types of parasol cells in primates: The ON-parasol ganglion cells respond with an increase in firing rate when stimulated by light in the centre of the receptive field and decreased light in the surrounding area. OFF cells have the opposite responses (Dacey and Lee, 1994). Parasol cells receive excitatory synapses from diffuse bipolar cells, which, in turn, receive input from several red and green cones. Two distinct types of diffuse bipolar cells (DB2 and DB3) provide input to OFF parasol cells while DB4 and DB5 are presynaptic to ON parasol cells (Jacoby et al., 1996; Jacoby, 1998). In synaptic connections between ON-centre parasol cells and other cells, ∼20% of the input is from bipolar cells and the remainder of the signal is introduced from amacrine cells, including AII and A17 cells (Jacoby et al., 1996). Rod photoreceptors synapse to rod bipolar cells that relay indirectly to the ON parasol cells through amacrine cells. Parasol RGCs play a role in motion and flicker perception and depth processing based on motion parallax (McMahon et al., 2004; Schiller, 2010).

#### 2.1.3 Large bistratified RGCs

Large bistratified cells resemble small bistratified cells but have consistently larger dendritic fields (Dacey et al., 2003). Preliminary findings suggest that their response characteristics resemble those of small bistratified cells (Dacey et al., 2003) which means that they may receive input from blue cone bipolar cells. Large bistratified cells receive not only S-cone ON-pathway input, but also (L + M) cone OFF-opponency (inhibitory) signals. However, neither their response properties nor their synaptic connectivity have been studied systematically (Dacey et al., 2003; Dacey and Packer, 2003; Kim et al., 2021; Patterson et al., 2020b; Peterson and Dacey, 2000). They project into the inferior pulvinar in the thalamus.

#### 2.1.4 Smooth monostratified RGCs

Smooth monostratified cells share many properties with parasol cells, including projections to the LGN and the SC. Two populations have been discovered; inner-ON and outer-OFF populations, with narrowly monostratified dendritic trees that were found to resemble the dendrites of parasol cells (Crook et al., 2008). Additionally, like parasol cells, smooth cells sum input from L- and M-cones, lack measurable S-cone input, display high spike discharge rates, and high contrast and temporal sensitivity (Crook et al., 2008; Rhoades et al., 2019). However, smooth cells can be uniquely distinguished from parasol cells by their smaller soma and intraretinal axon diameters. Smooth cells exhibit thick primary dendrites and a simple radiating branching structure with no spines and few short branchlets. By comparison, parasol cells in the same retinal locations, have more branched dendrites and a moderate density of spinelike structures and short branchlets (Crook et al., 2008; Rhoades et al., 2019). It has been suggested that smooth monostratified and parasol cells share presynaptic partners, but studies in marmoset have demonstrated that outer smooth monostratified, unlike outer parasol cells, do not show strong connectivity to DB3a cells (Masri et al., 2019).

#### 2.1.5 Small bistratified RGCs

This cell type accounts for ∼5–8% of primate RGCs (Masri et al., 2019) and they project to the koniocellular layers of the LGN (Crook et al., 2008; Dacey and Lee, 1994; Percival et al., 2014). Branches stratify in both layers (inner ON and outer OFF). The inner ON branches receive excitatory input from S-ON bipolar cells initiated by S-cones, while opposed (L + M)-OFF light responses arrive through outer OFF branches (Dacey et al., 1996). This arrangement is thought to provide good color vision with relatively low spatial resolution. Electron and light microscopic studies reveal that small bistratified cells receive inputs from bipolar and amacrine cells that relay to both the OFF and ON small bistratified cell dendritic trees (Calkins et al., 1998; Ghosh and Grünert, 1999; Percival et al., 2009). These studies also suggested that input to the outer tier involves the diffuse bipolar cells DB2 and DB3a (Masri et al., 2017). Bipolar input to the inner dendrites of small bistratified cells derives from blue cone bipolar cells (Calkins et al., 1998; Dacey and Lee, 1994; Ghosh et al., 1996; Wool et al., 2019). The amacrine type(s) providing input to the inner tier have not been identified but it is worth noting that the small bistratified cells are tracer-coupled to bistratified knotty 2 amacrine cells. The koniocellular layers not only receive input from small bistratified (blue ON/yellow OFF) cells (Crook et al., 2008; Dacey and Lee, 1994; Percival et al., 2009; Szmajda et al., 2008; Tailby et al., 2008), but also from multiple types of wide-field ganglion cells (Percival et al., 2011; Percival et al., 2014; Szmajda et al., 2008; Tailby et al., 2008). Wide-field ganglion cells (but not small bistratified cells) also project to the SC, presumably by axon collaterals in both macaque (Crook et al., 2014; McMahon et al., 2004; Rodieck and Watanabe, 1993) and marmoset (Grünert et al., 2021; Kwan et al., 2019).

#### 2.1.6 Recursive monostratified/bistratified RGCs

There are two types of recursive cells, the recursive monostratified cell (stratifying in the ON sublamina) and the recursive bistratified cell with dendrites in both the OFF and the ON sublamina of the IPL (Dacey, 2004; Masri et al., 2019; Moritoh et al., 2013). They have moderately densely branched dendritic trees in which many secondary branches tend to curve back towards the soma, and they play a role in direction selectivity in primates similar to the directionally selective, motion-sensitive RGCs (dsRGCs) of the rabbit and mouse (Dacey, 2004; Detwiler et al., 2019). Mono and bi-stratified RGCs connect to diverse types of starbursts amacrine cells (SACs) and bipolar cells. Bistratified cells connect to the ON-OFF SACs, while the monostratified cells connect to the on-SACs. The bipolar input to recursive bistratified cells includes DB3a, DB3b and DB2 cells (Masri et al., 2019), whereas the inner dendrites may receive input from DB4 and DB5 cells, but the circuitry of these cells has not been studied in detail. Also, bistratified cells project to the LGN and SC, while monostratified cells project to the accessory optic system (Dacey et al., 2019; Detwiler et al., 2019; Grünert et al., 2021).

#### 2.1.7 Thorny RGCs

There are three types of thorny RGCs in the primate retina that account for ∼1% of ganglion cells: ON-narrow thorny, OFF-narrow thorny, and broad thorny (Ghosh et al., 1996; Ghosh and Grünert, 1999; Grünert and Paul, 2020; Peterson and Dacey, 2000; Puller et al., 2015). All are sensitive to small moving stimuli and facilitate “catch-up” saccades during smooth eye movement. Recently, narrow thorny ganglion cells have been discovered to play a role in blue light vision (Mazzaferri et al., 2023). OFF-narrow thorny cells co-stratify with the OFF-starburst amacrine cells (ChAT) and bipolar cells (DB1 and DB2 cells) at the outer sublamina of the plexiform layer (Crook et al., 2014; Masri et al., 2019) while the ON-narrow and broad thorny cells co-stratify with the ON-amacrine and bipolar cells (DB6 cells) at the inner sublamina (Percival et al., 2014). Broad thorny cells, also named hedge cells (Ghosh et al., 1996) and T-group cells (Rodieck and Watanabe, 1993) stratify broadly in the middle of the IPL between the ChAT bands (Dacey et al., 2003; Masri et al., 2019; Percival et al., 2011; Puller et al., 2015). Most of their input comes through amacrine cells and only 2% comes from bipolar cells (Bordt et al., 2021). Broad thorny cells receive bipolar and amacrine input throughout their dendritic trees (Percival et al., 2011) and thus probably receive input from multiple OFF bipolar types, including DB2, DB3a, and DB3b (Masri et al., 2017) as well as from multiple ON bipolar types (DB4 and DB5). The amacrine input to broad thorny cells may derive from A1 amacrine cells, whose axons co-stratify with the dendrites of broad thorny ganglion cells. Davenport et al. (2007) suggested that A1 cells could create a strong suppressive field in broad thorny ganglion cells (Davenport et al., 2007). Other amacrine cells identified that connect to broad thorny ganglion cells include: two types of narrow-field cells (knotty bistratified Type 1 and wavy multistratified Type 2) (Bordt et al., 2021), two types of medium field amacrine cells (ON starburst and spiny), and three types of wide field amacrine cells (wiry Type 2, stellate wavy, and semilunar Type 2) (Bordt et al., 2021). Both broad thorny and narrow thorny cells project to the inferior pulvinar and SC (Grünert and Paul, 2020) and koniocellular layer of the LGN (Percival et al., 2014).

#### 2.1.8 Intrinsically photosensitive RGCs (ipRGCs)

In humans, the reported number of ipRGCs varies from ∼4,000 to more than 7,000, but it remains extremely marginal (0.4%–1.5%) compared to the 1.07 million ganglion cells in the human retina (Curcio and Allen, 1990; Esquiva et al., 2017; Hannibal et al., 2004; Hannibal et al., 2017; Morgia et al., 2010; Liao et al., 2016; Münch and Kawasaki, 2013; Mure, 2021). The fovea is devoid of ipRGCs (Mure, 2021). However, they are most abundant in the perifoveal region (∼15–40 cells/mm2) and their number declines to <5 cells/mm2 at 10 mm eccentricity and beyond (Hannibal et al., 2017; Liao et al., 2016; Nasir-Ahmad et al., 2019). These cells possess large, sparse dendritic fields. They are called intrinsically photosensitive because of their express the melanopsin photopigment, which enables phototransduction independently of rods and cones (Münch and Kawasaki, 2013). ipRGCs participate in contrast detection and play critical roles in non-image-forming vision, a set of light responses that include circadian entrainment, the pupillary light reflex (PLR), and the modulation of sleep/alertness, and mood (Mure, 2021). They also play a vital role during development, regulating lamination of cone photoreceptors, retinal vasculature, and the formation of retinogeniculate circuits (Raja et al., 2023).

In humans, four ipRGC subtypes (M1, M2, M3 and M4) have been defined (Hannibal et al., 2017) compared to six in rodents (Berry et al., 2023; Chen et al., 2021; Huang et al., 2023; Kiyama et al., 2025). They differ in dendritic arborization, expression levels of melanopsin, brain targets, and light responses. M1 ipRGCs have outer stratifying dendrites with a few smooth spines in the outer IPL, while M2 ipRGCs stratify in the inner IPL. M1 cells are subdivides into standard M1, gigantic M1 (GM1), displaced M1 (dM1), and gigantic dM1 (Esquiva et al., 2017; Hannibal et al., 2017; Liao et al., 2016; Nasir-Ahmad et al., 2019). M1 and GM1 both receive direct connections from rod bipolar cells (Hannibal et al., 2017). M1 cells connect with both ON (DB6) and OFF (DB1, DB2 and OFF midget) bipolar cells. Of note, in humans (but not mouse), dM1 cells constitute the majority of M1 population (Mure, 2021). Three types of OFF bipolar cells synapse onto displaced M1: diffuse bipolar DB1, DB2 and OFF midget bipolar cells, as well as rod bipolar cells (Bordt et al., 2022). dM1 also receive inputs from ON bipolar cells including: DB5, DB6, S-ON and ON-midget bipolar cells, and project to the dorsal LGN (Liao et al., 2016; Lima et al., 2012). They receive input from amacrine cells, including the dopaminergic type (Bordt et al., 2022). M2 ipRGCs have larger soma and more branched dendrites than M1 ipRGCs. M1 ipRGCs are reported to receive inhibitory input from short-wave cones via S-cone amacrine cells (dopaminergic amacrine cell) (Patterson et al., 2020b), whereas M2 ipRGCs receive input from S-ON bipolar cells and contribute to the blue cone pathway (Patterson et al., 2020b). In humans, M3 ipRGCs are found in the inferior and nasal part of the retina, with their soma located in the GCL and dendritic processes terminating in both S1 and S5 of the IPL (Hannibal et al., 2017). M4 are characterized by their large somas, weak melanopsin immunostaining, location in the inner IPL layer, and direct synapsing with rod bipolar cells (Hannibal et al., 2017). Dopaminergic amacrine cells make direct connection with M1 and dM1 cells, while GABAergic and AII amacrine cells synapse with M1, GM1, M2, and M4 cells (Bordt et al., 2022; Hannibal et al., 2017). Functionally, M1 ipRGCs project to the suprachiasmatic nucleus to synchronize the circadian clock (Hannibal et al., 2014), while M2, M3, and M4 ipRGCs project to the SC (Schmidt et al., 2011a; Schmidt et al., 2011b; Zhao et al., 2014) as well as to the dLGN, pretectal olivary nucleus (OPN) in the thalamus to control the pupillary response (Bordt et al., 2022; Hannibal et al., 2014).

### 2.2 Newly discovered human RGC subtypes

Recently, an ON-DSGC was discovered in the macaque retina similar to ON-DSGCs in other mammals and to the recursive monostratified RGCs described previously in macaque and marmoset retinas (Wang et al., 2023). Wang et al. (2023) found ON-type direction-selective ganglion cells (ON-DSGCs) in the macaque retina with a previously unknown mechanism for stabilizing gaze in primates. Using single-cell RNA transcriptomics, two-photon calcium imaging, and morphology, they confirmed the presence of ON-DSGCs, which encode image motion direction and project to brainstem nuclei to regulate compensatory eye movements to reduce image blur. These cells exhibit conserved molecular, morphological, and GABA-dependent direction-selectivity mechanisms that are common to non-primate mammals, which challenges the former hypothesis that cortical regions are primarily responsible for this reflex in primates. This discovery puts the retina at the center of visual processing in the primate, setting up a multimodal analysis for other types of RGCs and showing that primate vision is more similar to that of other mammals than previously thought.

### 2.3 Conserved RGC subtypes across mammalian species

Recent cross-species transcriptomic and functional analyses indicate a conserved set of RGC orthotypes across mammalian evolution despite differences in total subtype numbers between species. These observations include ipRGCs involved in non-image-forming functions, as well as molecular orthotypes linking mouse alpha RGCs to primate midget (sustained alpha) and parasol systems (transient alpha), and primate monostratified and bistratified RGCs to mammalian ON-DS and ON-OFF direction-selective circuits (ooDSGCs) respectively (Budoff and Poleg-Polsky, 2025; Tapia et al., 2022; Hahn et al., 2023; Huang et al., 2024). These findings suggest that primates, while specialized for high-resolution vision, maintain an “ancient” scaffold of RGC types for fundamental visual tasks like motion detection and circadian photoentrainment.

### 2.4 Human RGC subtype marker expression

RGCs are a phenotypically diverse groups of neurons and their characterization is based on the detection of molecular markers specific to different subtypes. However, many of these RGC subtype markers, such as transcription factors, cell surface molecules, and calcium-binding molecules, are often non-specific as individual markers will label multiple subtypes. Furthermore, marker expression is not always conserved across species, which complicates comparative studies. For instance, a mouse RGC subtype-selective marker might not define the same subtype in primates or other mammals. Such cross-species variation, combined with overlapping marker expression, hinders accurate classification of RGCs and inference of functional roles. Consequently, integrated approaches involving transcriptomics and morphology are necessary to streamline subtype identification. Studying mouse RGC subtypes can guide the search for novel human subtype-specific markers (Huang et al., 2024; Lu et al., 2020; Hahn et al., 2023). For example,: insights from mouse RGC subtypes such as their identification via scRNA-seq can inform human stem cell differentiation by highlighting conserved transcription factors (e.g., *ATOH7* for progenitor commitment, *POU4F2*/*BRN3B* for maturation). These insights guide protocols in hPSC-derived organoids or 2D cultures to mimic retinogenesis and generate subtype analogs. This knowledge is critical for optimizing *in vitro* hPSC differentiation protocols, which often lack distinct human-specific markers (e.g., limited subtype-specific antibodies for rare primate types). Analysis of mouse orthologs also enables cross-species comparisons through integrated transcriptomics and morphology, as demonstrated in multi-species atlases that reveal evolutionary divergences like primate foveal specialization. These comparisons allow for adaptation of mouse-derived strategies, such as CRISPR editing for resilience genes (e.g., Osteopontin/mTOR pathways from mouse optic nerve crush models (ONC)) to enhance resilience in human ipRGC-like cells. This approach not only optimizes *in vitro* hPSC protocols for subtype enrichment but also bridges translational gaps. However, challenges like non-conserved expression (e.g., mouse-specific markers not being conserved in human cells) necessitate human-focused multi-omics to refine these differentiation mechanisms. Here we review current molecular subtype markers for human and primate RGCs. (See Table 2 for comprehensive marker details).

**TABLE 2:** Human RGC subtype molecular markers.

RGC subtype	Markers	References
ON midget	*TPBG, GUCY1A3, MAP3K1, EOMES, RBPMS*	Peng et al. (2019); Yan et al. (2020)
OFF midget	*TBR, GUCY1A3, MAP3K1, SIX6, MEIS2, RBPMS*	Peng et al. (2019); Yan et al. (2020)
ON parasol	*CHRNA2, SPP1* *RBPMS2, MAP3K1, SNCG, SMI-32, THY1*	Peng et al. (2019); Yan et al. (2020); Krieger et al. (2017); Duan et al. (2015); Rheaume et al. (2018)
OFF parasol cells	CA8, *SPP1, RBPMS2, MAP3K1, SNCG, SMI-32, THY1*	Peng et al. (2019); Yan et al. (2020); Krieger et al. (2017); Duan et al. (2015); Rheaume et al. (2018)
Small bistratified	*CALB1-2*	Lee et al. (2016)
Large bistratified	*CALB1-2, SATB2*	Lee et al. (2016)
Broad thorny ON-OFF	*CALB1-2, CAMKII, SATB2, RBPMS*	Hendry and Yoshioka (1994); Sincich et al. (2004); Callaway (2005); Chandra et al. (2017); Baldicano et al. (2022); Nasir-Ahmad et al. (2021)
Narrow thorny ON	*CALB1-2*	Chandra et al. (2017)
Narrow thorny OFF	*CALB1-2, SATB2*	Chandra et al. (2017); Nasir-Ahmad et al. (2021)
Recursive bistratified ON-OFF	*SATB1, SATB2, RBPMS*	Chandra et al. (2019); Nasir-Ahmad et al. (2021)
Intrinsically photosensitive RGCs (ipRGCs)	*OPN4, SATB2, CAMKII, CALB1-2, SNCG, SMI-32, SPP1*	Santina and Ou (2017); Hendry and Yoshioka (1994); Sincich et al. (2004); Callaway (2005); Dacey et al. (2005); Hannibal et al. (2014); Jusuf et al. (2007); Liao et al. (2016); Nasir-Ahmad et al. (2019); Peng et al. (2019); Nasir-Ahmad et al. (2021); Dhande et al. (2019); Chandra et al. (2017); Hannibal et al. (2004); Chandra et al. (2019)

Peng et al. (2019) identified markers for various human subtypes. They found that *TBR1* is expressed in OFF Midget cells, *TPBG* in ON Midget cells (Peng et al., 2019; Yan et al., 2020), *RBPMS2* was found to be expressed in both ON and OFF midget cell subtypes (Yan et al., 2020), *EOMES* by ON midget (Peng et al., 2019) and *MEIS2* by the OFF-midget cells (Peng et al., 2019). *CHRNA2* was found to be expressed in ON Parasol cells (Peng et al., 2019; Yan et al., 2020) and CA8 in OFF Parasol cells (Peng et al., 2019). *SPP1* and *RBPMS2* are expressed by both Parasol cell types and *GUCY1A3* by both Midget cell types (Peng et al., 2019). The same group found that *MAP3K1* is expressed by both midget and parasol cells and *SIX6* by midget cells (Peng et al., 2019).

The calcium binding protein Calretinin, also known as calbindin 2 (*CALB2*) (formerly 29 kDa calbindin) (Diamond et al., 1993; Goodchild and Martin, 1998; Jones and Hendry, 1989; Solomon, 2002) as well as the alpha subunit of calcium-/calmodulin-dependent proteinkinaseII (*CaMKII*) (Callaway, 2005; Hendry and Yoshioka, 1994; Sincich et al., 2004) are expressed in macaques, humans and marmosets. These include a variety of wide-field ganglion cells including the ipRGCs (Chandra et al., 2019), broad thorny cells and narrow thorny cells (Chandra et al., 2017) and in both small and large bistratified cells (Lee et al., 2016). Notably, all melanopsin-expressing cells are also CaMKII-positive.

In mice, the transcription factor Satb2 is expressed in three RGC types: oo-DSGCs, OFF-DSGCs, and an OFF-sustained RGC type. In contrast, in macaque, human, and marmoset retinas, *Satb2*-positive cells constitute only 1.5%–4% of the RGC population, with a slight increase from central to peripheral regions. In macaque and human retinas, over 80% of *Satb2*-expressing cells are inner and outer stratifying ipRGCs, while in marmosets, over 60% are broad thorny cells, with smaller proportions being recursive bistratified, large bistratified, and outer stratifying narrow thorny cells. All Satb2-positive cells also express RBPMS, a general RGC marker. Additionally, *Satb2*, along with *Camk2*, is expressed in broad thorny, OFF-narrow thorny, ON-ipRGCs, OFF-ipRGCs, and large bistratified cells, while ON-OFF recursive bistratified cells express both *Satb1* and *Satb2*. The *Opn4* gene, encoding melanopsin, is expressed in ipRGCs across species, with detectable levels in some peripheral RGC clusters. In humans, higher melanopsin expression is linked to M1 ipRGCs, while other subtypes (M2–M4) show lower expression or are too rare to detect, highlighting variability in marker expression across species and RGC subtypes.

In summary, these species expression variations, combined with overlapping marker profiles, necessitate integrated approaches like transcriptomics and morphology to improve RGC subtype identification and understand their functional roles.

## 3 RGC subtype vulnerability to stress and injury

**FIGURE 3:**
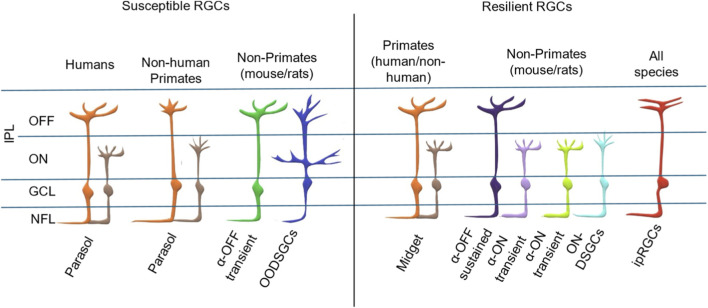
Resilient and susceptible RGC subtypes and their dendritic stratifications. A cartoon showing dendritic stratifications into the IPL (with sublaminae divided into ON and OFF). Human, non-human primate, and non-primate susceptible RGC subtypes are depicted on the left, and resilient subtypes are displayed on the right.

According to many published studies, RGCs are particularly vulnerable neurons in the retina, and their degeneration is the immediate cause of vision loss in a variety of retinal diseases, such as glaucoma and ischemic optic neuropathies (Alqawlaq et al., 2019; Soucy et al., 2023). Yet, there is controversy in the field as to whether certain RGC subtypes are more or less vulnerable to injury (Santina and Ou, 2017) (Figure 3). Questions have been raised as to whether soma size (small vs. large), stratification (ON vs. OFF), firing (sustained vs. transient) and location (central vs. peripheral) affect the susceptibility of RGCs to injury. This applies to glaucoma and a various optic nerve injury model, including exposure to elevated intraocular pressure (IOP), ONC or transection, and ischemic or excitotoxic stresses. Answering these questions will help researchers overcome current challenges in engineering cells with increased resilience and protecting the more vulnerable subtypes. This strategy can be applied to future efforts to preserve vision and prevent blindness.

### 3.1 Human studies

Functional and physiological testing in glaucoma has revealed a decrease in the activity of parasol cells (magnocellular pathway) in the visual cortex. Using stimuli that target the magnocellular pathway in patients with open-angle glaucoma or ocular hypertension, Howe and Mitchell (1992) found a reduction in the visually evoked potential (VEP) response amplitudes and contrast sensitivity (Howe and Mitchell, 1992). Furthermore, by separating the magnocellular and parvocellular components of the VEP, Klistorner and Graham (1999) identified a reduction in the magnocellular component in those with early glaucoma, while responses in the parvocellular component declined in those with more severe glaucoma (Klistorner and Graham, 1999). In a *postmortem* study of the LGN from glaucoma patients, the mean parasol cell density was significantly lower than in the control group, whereas no significant difference was found in the midget cell density (Chaturvedi et al., 1993). Taken together, these findings suggest that parasol cells are more susceptible to IOP-induced injury than midget cells.

Clinical testing has also supported this observation. Patterson et al. (2020a) found that visual acuity was significantly worse in patients responding to high temporal frequency light stimuli (a test that targets the magnocellular pathway) compared to stationary stimuli targeting the parvocellular pathway (Patterson et al., 2020a). Similarly, Sun et al. (2008) observed reduced contrast sensitivity in the magnocellular pathway of glaucoma patients, while responses favoring the smaller RGCs of the parvocellular pathway were unaffected (Sun et al., 2008). Using fMRI, Zhang and colleagues (2016) found that early-stage glaucoma patients were less responsive to transient achromatic stimuli than to sustained chromatic stimuli in the magnocellular layers of the LGN and the superficial layer of the SC, but this was not observed in the parvocellular layers or cortical visual areas (Zhang et al., 2016). They concluded that early-stage glaucoma causes selective functional loss of the larger cells in the human LGN and SC.

### 3.2 Non-human primate studies

Earlier work in non-human primates and human tissue supported the concept that RGCs with the largest cell bodies and axons are the most susceptible to injury (Glovinsky et al., 1991; Quigley, 1999; Quigley et al., 1987; Quigley et al., 1988). To better understand the effects of IOP elevation in a comparable species, several studies have modelled glaucoma by laser-treating the trabecular meshwork of non-human primates. This treatment impedes aqueous outflow, leading to increased IOP, a major risk factor for glaucoma (Alqawlaq et al., 2019). Like humans, primates possess a lamina cribrosa and can closely model the effects of elevated IOP on the biomechanics of the human optic nerve head. Applying this method to cynomolgus monkeys, scientists found fewer RGCs with large somata and fewer large-diameter axons in the optic nerve (Glovinsky et al., 1991). An immunohistochemical study showed that these RGCs had reduced neurofilament staining, which is indicative of large RGC types (Vickers et al., 1995). Furthermore, parasol RGCs have exhibited subtle shrinkage of their somata, axons and dendritic fields before observing changes were observed in midget RGCs (Weber et al., 1998). Compared to normal optic nerves, glaucomatous optic nerves showed a greater loss of large diameter axons. Subsequent work examining RGC size and rates of cell death in the whole-mount retina suggested a greater reduction of larger-diameter RGCs, which may correlate with larger-diameter axons. (Davis et al., 2020; Santina and Ou, 2017; Kerrigan-Baumrind et al., 2000). Finally, research has shown that axonal transport to the magnocellular layers was more impaired than to the parvocellular layers of the dorsal lateral LGN in non-human primates with chronic IOP elevation (Dandona et al., 1991).

### 3.3 Comparisons to non-primate models

Work in mouse and rat glaucoma models has supported the idea that RGCs with large somata (i.e.,; alpha RGCs) are more vulnerable to injury (De et al., 2025; Glovinsky et al., 1991; Kerrigan-Baumrind et al., 2000; Quigley, 1999; Quigley et al., 1987; Quigley et al., 1988; Vickers et al., 1995). In comparison, ipRGCs have large dendritic fields and thus might expected could be susceptible to injury. However, this RGC type appears resistant to injury in terms of both total cell loss and dendritic complexity (Li et al., 2006). Therefore, data obtained from primates (human and non-human) as well as non-primates show consistent results supporting the hypothesis that RGCs with large diameter somata are lost before small ones in glaucoma models. However, the underlying explanation for this difference remains unclear.

Stratification to the outer and inner sublaminae also affects the degree of RGC susceptibility to injury. RGCs with most of their dendrites in the OFF sublamina undergo the greatest morphological change, whereas those RGCs with most dendrites in the ON sublamina remain resistant to IOP elevation (Fu and David, 2010; Salinas-Navarro et al., 2009). Many studies have also shown that the proportion of OFF transient RGCs lost is greater than for ON sustained RGCs (Santina and Ou, 2017; Duan et al., 2015; Quigley, 1999; Tran et al., 2019). The ooDSGCs stratify to both the ON and OFF sublaminae, but only dendrites in the OFF layers were lost in a mouse glaucoma models (Bray et al., 2019). Since ooDSGCs have transient responses, these findings are also consistent with the higher vulnerability exhibited by transient-responding RGCs compared to sustained-responding RGCs (El-Danaf and Huberman, 2015). Similar to the ooDSGCs in non-primates, the recursive monostratified and bistratified RGCs play a role in the direction selectivity in primates similar to the directionally selective, motion-sensitive RGCs (dsRGCs) of the rabbit (Detwiler et al., 2019; McMahon et al., 2004). We have found no study to date that has investigated the recursive cell count in glaucoma models or stem cell culture studies. Therefore, it seems worthwhile to assess molecular markers that can be used to identify the susceptibility of this cell type in future studies.

Differences between RGCs in receptor expression, metabolic usage, or external vascular and biomechanical environment may help to explain RGC subtype susceptibility to IOP-induced injury. Several mechanisms may contribute to this vulnerability, including differences in the expression of *PANX1*, *P2X7*, *AMPA*, and transient receptor potential vanilloid receptors in the transient OFF alpha RGCs (Dvoriantchikova et al., 2018; Locovei et al., 2007). In glaucoma, ATP released from dead cells activates these receptors, leading to *Ca* ^2+^ influx and subsequent cell death (Ryskamp et al., 2011; Sappington et al., 2009). Another factor might be the relative proximity of RGCs and their dendrites to blood supply in the IPL (El-Danaf and Huberman, 2015; Ivanova et al., 2014; Nimkar et al., 2025) or differences in metabolic requirements. OFF RGCs are reported to be more active, having greater energy demands, and may thus be at greater risk during stress (Wang A. Y. et al., 2020). Such differences may make certain RGCs more sensitive to IOP elevation and its associated biomechanical and vascular stress. Additionally, RGC survival has been found to be variable across species (De et al., 2025).

Of note, ipRGCs consistently demonstrate a higher survival ability in certain pathological and experimental conditions. In the mouse, ipRGCs appear more resistant than other RGCs to various insults, including optic nerve injury, glutamate-induced excitotoxicity, and early-stage glaucoma (Cui et al., 2015; Tran et al., 2019). In human patients, ipRGCs resist neurodegeneration in two inherited mitochondrial disorders that cause blindness: Leber hereditary optic neuropathy and dominant optic atrophy (Morgia et al., 2010). This ability is independent from melanopsin expression, as ipRGC resilience is preserved in a mouse models bearing the mutation causing dominant optic atrophy even when lacking melanopsin (González-Menéndez et al., 2015). Specific metabolic properties, such as higher mitochondrial activity or content, have been hypothesized as potential protective mechanism. However, the reason ipRGCs are resistant to injury is still not well understood. Although ipRGCs show relative resistance, their low abundance (∼1% of RGCs) limits clinical utility, whereas midget RGCs (70%–80%) and parasol RGCs (10%) are prioritized for therapy due to their essential roles in high-acuity color vision, detail resolution, and motion/contrast sensitivity in primates. Differential vulnerabilities in glaucoma such as the early susceptibility of parasol RGCs due to their large somata, thick axons, and high metabolism support targeted neuroprotection against calcium dysregulation or mitochondrial issues. Conversely, midget RGCs’ thinner axons and high energy demands favor regenerative approaches like stem cell transplantation. Meanwhile, ipRGC resilience enables optogenetic strategies for non-image-forming functions like circadian regulation in advanced disease, emphasizing precision medicine through subtype-enriched organoids for drug screening and CRISPR editing to enhance survival.

Ultimately, understanding species similarities and differences in RGC subtype specific gene expression will facilitate the discovery of new markers. This will enable the design of protocols to protect vulnerable subtypes and enhance resistance to injury.

## 4 *In vitro* models of human RGC subtypes

**FIGURE 4:**
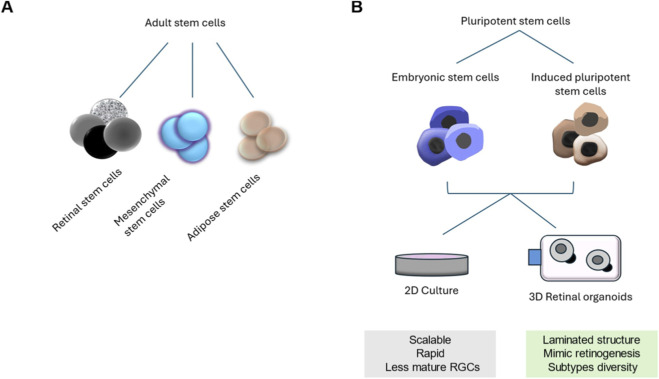
Strategies for *in vitro* RGC differentiation. **(A)** Adult stem cells can be conveniently isolated from different tissues and organs, including the eye’s pigmented ciliary epithelium (retinal stem cells). Attempts to derive RGCs from alternative stem cell populations including adipose tissue (adipose stem cells and mesenchymal stem cells), bone marrow and periodontal ligament (mesenchymal stem cells have met with varying success but often hindered by limited subtype diversity and insufficient axonal outgrowth). **(B)** In comparison, pluripotent stem cells are derived either from embryonic stem cells or induced pluripotent stem cells. Cells from adult or pluripotent sources can be differentiated into RGCs through culturing in stationary (adherent) 2D culture or in 3D suspension culture (retinal organoids).

In the human eye, lost or damaged RGCs cannot be replaced endogenously, as the stem cells that build the retina during development enter a state of quiescence after birth (Tropepe et al., 2000). Several methods have been proposed to generate new RGCs, including the use of autologous adult retinal stem cells (Tropepe et al., 2000; Coles et al., 2004; Cho et al., 2012). In addition, multiple studies have attempted to differentiate RGCs from other extraretinal stem cell populations, with varying degrees of success. These efforts include adult mesenchymal stem cells (MSCs) (Eraslan et al., 2023; Dodina et al., 2024), autologous adipose-derived stem cells (Rezanejad et al., 2014), autologous bone marrow derived stem cells and Müller glia (MG) cells (Cen and Ng, 2018; Silva-Junior et al., 2021; Soucy et al., 2023). However, these studies frequently utilize rodent models and highlight paracrine-mediated neuroprotection rather than the robust generation of functional human RGC subtypes. Although MSCs and MG (Silva-Junior et al., 2021; Soucy et al., 2023) offer potential therapeutic avenues, their capacity to recapitulate the complex diversity of the human RGC landscape remains limited. Consequently, human PSCs including ESCs and iPSCs, have emerged as the gold standard for modeling human RGC subtype specification and functional maturation (Kosior-Jarecka and Grzybowski, 2024; Zhang et al., 2021) (Figure 4). Pluripotent stem cells give rise to a broader range of cell types compared to adult stem cells, making them preferred source for many scientists.

Many previous studies focused on growing RGC subtypes *in vitro* have relied upon animal models, which have enabled the identification of these cells and the study their functional characteristics (Dhande et al., 2015; Marcucci et al., 2016; Sanes and Masland, 2015). However, comparably few studies have been performed in humans due to the limited availability of adult tissue and the inaccessibility of the human retina at early developmental stages.

hPSCs provide a powerful tool for developmental studies because they can self-renew and give rise to all cell types of the body (Takahashi et al., 2007; Trimarchi et al., 2007; 2008; Yu et al., 2007). Many previous efforts have examined the ability of hPSCs to give rise to RGCs (Fligor et al., 2018; Gill et al., 2016; Lei et al., 2024; Luo and Chang, 2024; Ohlemacher et al., 2016; Riazifar et al., 2014; Sluch et al., 2015; Tanaka et al., 2016; Teotia et al., 2017). However, this differentiation has primarily focused on the generation of general RGCs without addressing the numerous subtypes that exist. RGC differentiation strategies from hPSCs generally fall into three primary categories: small molecule-directed 2D differentiation, 3D retinal organoid differentiation, and direct transcription factor-driven differentiation.

Small molecule-directed 2D RGC differentiation protocols commonly work by directing hPSCs toward a neuroectodermal fate through the dual inhibition of BMP and TGF-β signaling (e.g., using drugs like dorsomorphin or SB431542). This is followed by retinal progenitor specification using FGF2 and IGF1 to promote the formation of optic vesicle-like structures. Subsequent RGC commitment is achieved through Notch inhibition and the expression of proneural factors such as *ATOH7* (the human ortholog of *Math5*), which drives cell cycle exit. Maturation is further guided by POU-domain transcription factors, particularly *BRN3B* and *ISL1*, which are essential for axonal outgrowth and terminal differentiation (Gill et al., 2014; Ji and Tang, 2019; Sluch et al., 2015; Lee et al., 2018; Luo et al., 2022).

3D retinal organoid differentiation leverages the intrinsic self-organizing capacity of hPSCs in suspension culture to form laminated, optic-vesicle-like structures. Within this 3D architecture, RGCs emerge spontaneously, offering superior biological complexity and mimicry of *in vivo* retinogenesis and subtype diversity. Modern protocols can achieve a stratified retinal structure over several months, providing a more physiologically relevant model than 2D system (Fligor et al., 2018).

Finally, the direct transcription factor-driven differentiation approach involves the exogenous, forced expression of key proneural and POU-domain transcription factors (e.g., *BRN3B*, or *ISL1*) to bypass traditional developmental stages. This method rapidly convert hPSCs or somatic cells directly into RGC-like neurons, often within one to 2 weeks, which is significantly faster than the organoid-based methods (Agarwal et al., 2023; Costea et al., 2025; Wang J. et al., 2020).

Each system offers distinct advantages for differentiating hPSCs into RGCs. Cultures in 2D provide simplicity, scalability, and precise control over signaling cues, facilitating higher RGC yields of RGC progenitors and easier downstream analyses like immunopurification. However, they often lack the physiological architecture and mature synaptic connectivity found *in vivo*, resulting in reduced cell-cell interactions and limited mimicry of retinal lamination (Oswald and Petr, 2018). In contrast, 3D retinal organoids better recapitulate *in vivo* retinogenesis, promoting self-organization into laminated structures with improved RGC maturation, synaptic formation, and subtype diversity but faces challenges such as high variability, reproducibility, long differentiation timelines, axon growth, and nutrient diffusion limits in the absence of vascularization (Wang et al., 2025; Harkin et al., 2024). Markers expression also varies widely between the two systems. In 2D cultures, small molecule-directed differentiation enables rapid induction of markers like *BRN3B* within 4–6 weeks (Gudiseva et al., 2021) and high *THY1* expression for efficient isolation, though *RBPMS* is often lower or delayed (Chavali et al., 2020). In contrast, 3D organoids more closely mimic *in vivo* retinogenesis with sequential marker emergence *ATOH7* in progenitors, followed by *BRN3B*/*ISL1* and later *RBPMS*/neurofilaments. Yet *THY1* remains low unless enriched via dissociation and 2D replating (Aparicio et al., 2017; Rabesandratana et al., 2020). Hybrid approaches, such as transitioning from 3D to 2D for RGC enrichment, may optimize outcomes for research and therapeutic applications (Li et al., 2021; Luo et al., 2022).

However, while pluripotent stem cell-derived RGCs offer promising systems for disease modeling and regenerative therapies, several important limitations persist. These limitations include incomplete maturation, where derived RGCs often exhibit immature phenotypes with limited axonal outgrowth. Also, incomplete synaptic connectivity, and poor electrophysiological responses have been reported compared to native cells (Singh and Nasonkin, 2020; Kurzawa-Akanbi et al., 2024). Heterogeneity in differentiation efficiency leads to mixed populations, complicating subtype-specific studies, and the absence of vascular and immune components *in vitro* restricts modeling of complex pathologies (Kurzawa-Akanbi et al., 2024). Post-transplantation challenges, such as poor integration, immune rejection, and limited long-term survival (e.g., up to 4 months in some models), further hinder clinical translation (Lei et al., 2024; Yang et al., 2021).

To move beyond heterogeneous pan-RGC populations, recent strategies have focused on manipulating the transcriptional landscape. Beyond the core *ATOH7*/*BRN3B*/*ISL1* axis that drives general RGC commitment, researchers are now targeting specific factors such as SoxC (*Sox4*, *11*, *12*) and GDF signaling to direct subtype-specific fates or to bias cells toward specific projection identities (Kuwajima et al., 2017; Norsworthy et al., 2017; Enriquez et al., 2025). Furthermore, the functional maturation of these *in vitro* models is limited by the absence of the native microenvironment. To overcome this ‘maturation bottleneck,’ approaches have started to be integrated into differentiation workflows. These methods include **c**o-culture with astrocytes or microglia to enhance synaptic pruning and ionic current maturation (VanderWall et al., 2019), the development of retinal-thalamic assembloids to provide RGCs with their physiological axonal targets (Fligor et al., 2021), and the application of extracellular matrix (ECM) scaffolds or mechanical patterning to sustain long-term culture (Gomes et al., 2025). Additionally, chronic electrical stimulation has been shown to improve the electrophysiological excitability of RGCs, bringing their functional profiles closer to their adult *in vivo* counterparts (Huberman et al., 2008). By shifting from pan-RGC generation to these subtype-targeted and maturation-enhanced protocols, *in vitro* models are becoming increasingly relevant for studying the subtype-specific vulnerability observed in diseases like glaucoma.

The ability to more accurately generate these cells from hPSCs, allows for the study of the cellular mosaicism that exists among RGCs of the human retina, with important implications for how these subtypes differ in their functionality as well as how they may be affected in disease states.

## 5 RGC subtypes in human retinal organoid cultures

Retinal organoids are three-dimensional structure derived from stem cells that recapitulate the temporal development and spatial lamination of the *in vivo* retina. They display characteristic stratification, with RGCs located in inner layers and photoreceptors in the outer peripheral layers. While many studies have utilized organoids to study normal histology, physiology and diseases of the outer retina including photoreceptors and RPE (Phillips et al., 2012; Völkner et al., 2016; Wahlin et al., 2017; Zhong and Gutierrez, 2014), fewer have focused specifically on RGC development (Ohlemacher et al., 2019). Because RGCs are among the first cell types to develop, they provide a more feasible research timeline than that of photoreceptors, which can take over 200 days to mature. Several protocols have been developed to enrich organoids for RGCs using various strategies (Freude et al., 2020; Gill et al., 2014; Gill et al., 2016; Rabesandratana et al., 2020; Reichman et al., 2017; Soucy et al., 2023; Tanaka et al., 2016; Zhu et al., 2018). However, a recurring limitation in these studies is the focus on pan-RGC markers rather than specific subtype identification. Examples of most used protocols are briefly summarized in the following paragraphs.

Zhu et al. (2018): This protocol treated hPSCs with BMP, Wnt, blockers, supplemented with IGF1 for 5–6 days during maintenance passages, to facilitate retinal induction (Zhu et al., 2018). While the resulting 6- and 12-week organoids expressed *BRN3* and *ISL1*, no absolute cell counts were performed to assess the fold increase in RGCs, and no subtype-specific markers were utilized.

Ohlemacher et al. (2016) & Langer et al. (2018): These researchers found that RGCs expressed RGC-associated markers that reached high levels by day 50 in cultures (about 8–12 times compared to the expression level at day 25), including: *ATOH7*, *PAX6*, *BRN3B*, *ISL1*, *RBPMS*, *SNCG*, and *OPSIN4*. By day 70, these RGCs expressed *MAP2* in somatodendritic regions and *TAU* in axonal extensions (Ohlemacher et al., 2016; Langer et al., 2018). While successful in enriching the general RGC population, this protocol did not differentiate between specific subtypes.

Tanaka et al. (2015): Using a modified protocol adapted from Nakano et al. (2012) without BMP, Wnt, Notch, TGFB blockers (Tanaka et al., 2015). This group observed a 30-fold increase in the expression of *BRN3B*, *ATOH7*, *ISL1*, *SNCG*, and *TUJ1* by day 34 (Tanaka et al., 2015). Again, the analysis remained at the pan-RGC level.

Freude et al. (2020): This study explored the theory that enriching for MG could support RGC growth. The authors identified MG via *GFAP*, *RLBP1*, and *CD44* expression, while RGCs were identified using *BRN3A*. Although they used magnetic-activated cell sorting (MAC) to isolate RGC progenitors (expressing *CHX10* and Nestin), the study did not employ markers to distinguish between RGC subtypes.

Reichman et al. (2017) & Rabesandratana et al. (2020): These studies utilized a chemically defined E6 medium followed by ProB27 medium supplemented with FGF2 (Reichman et al., 2017). They observed that while *BRN3A*, *RBPMS*, and *HuC/D* were present between days 56 and 84, RGC density decreased by day 98, a common “loss of RGCs” phenomenon in aging organoids. To counter this, they dissociated day-56 organoids and replated them in 2D culture, which significantly enhanced RGC survival and maturation. Flow cytometry confirmed that 60% of the resulting cells were *THY1*-positive RGCs (Rabesandratana et al., 2020).

Kim et al. (2023): This group utilized a pro-neural induction medium and transitioned manually isolated optic vesicles to 3D suspension at day 25, adding retinoic acid at day 42. RNA-seq confirmed *THY1* expression (Kim et al., 2023). Similarly, organoids grown from glaucoma-patient iPSCs (e.g., OPTN E50K mutants) demonstrated increased apoptosis and impaired axonal transport (VanderWall et al., 2020). These models are vital for drug screening but currently generate heterogeneous RGC populations without specific subtype enrichment (Daniszewski et al., 2022; Hameed and Sharma, 2025).

In summary, all the above-mentioned retinal organoids grown from PSCs derived from either healthy or diseased individuals never elaborated on the importance of enriching specific subtypes nor were they developed with this idea in mind. There remains a significant gap in the field regarding the directed differentiation of high-acuity midget cells, motion-sensitive parasol cells, or specialized types like recursive RGCs. Future studies should address this gap by developing protocols that utilize subtype-specific molecular markers and transcription factor biasing. This will enable more precise investigations into the pathogenesis of glaucoma and facilitate the development of targeted therapeutic interventions for the most vulnerable RGC subtypes.

## 6 Conclusion

In contrast to other retinal neurons, RGCs are a diverse population, with many distinct subtypes identified in varied species, including humans. Each subtype is associated with characteristic topography, functionality, and responses to disease. To replace RGCs lost during disease or injury, fully characterized human-derived stem cell culture and differentiation protocols need to be established for each subtype. However, the identification, characterization, and culture of RGC subtypes in humans still faces many challenges. The development of this field will depend on an understanding of how each subtype is generated during development, and the discovery of new selective markers, particularly for vulnerable subtypes. This knowledge will facilitate efforts to preserve subtype function and survival, and to enrich and manipulate them in cell rescue and replacement therapies.
